# Impact of Smartphone App–Based Psychological Interventions for Reducing Depressive Symptoms in People With Depression: Systematic Literature Review and Meta-analysis of Randomized Controlled Trials

**DOI:** 10.2196/29621

**Published:** 2022-01-27

**Authors:** Maria J Serrano-Ripoll, Rocío Zamanillo-Campos, Maria A Fiol-DeRoque, Adoración Castro, Ignacio Ricci-Cabello

**Affiliations:** 1 Health Research Institute of the Balearic Islands (IdISBa) Palma de Mallorca Spain; 2 Balearic Islands Health Services, Primary Care Research Unit of Mallorca Palma de Mallorca Spain; 3 Primary Care Prevention and Health Promotion Network RedIAPP Barcelona Spain; 4 Research Institute of Health Sciences (IUNICS) University of the Balearic Islands Palma de Mallorca Spain; 5 Centro de Investigación Biomedica en Red (CIBERESP) Madrid Spain

**Keywords:** smartphone technology, mental health interventions, depression, eHealth, mHealth, apps, systematic review, meta-analysis, mobile phone

## Abstract

**Background:**

Depression is a serious, disabling mental disorder that severely affects quality of life. Patients with depression often do not receive adequate treatment. App-based psychotherapy is considered to have great potential to treat depression owing to its reach and easy accessibility.

**Objective:**

We aim to analyze the impact of app-based psychological interventions for reducing depressive symptoms in people with depression.

**Methods:**

We conducted a systematic literature review and meta-analysis. We searched Medline, Embase, PsycINFO, Web of Science, and Cochrane Central Register of Controlled Trials from inception to December 23, 2020. We selected randomized controlled trials to examine the impact of app-based psychological interventions for reducing depressive symptoms in people with depression. Study selection, data extraction, and critical appraisal (using the Cochrane Risk of Bias tool for randomized studies and the ROBINS-I tool for nonrandomized studies) were conducted independently by 2 reviewers. Where possible, we pooled data using random effects meta-analyses to obtain estimates of the effect size of the intervention. We conducted post hoc meta-regression analyses to explore the factors associated with intervention success.

**Results:**

After screening 3468 unique references retrieved from bibliographic searches and assessing the eligibility of 79 full texts, we identified 12 trials (2859 participants) evaluating 14 different interventions. Of 14 trials, 7 (58%) were conducted in the United States; 3 (25%) trials, in Asia (Japan, South Korea, and China); 1 (8%) trial, in Australia; and 1 (8%) trial, in Germany. Of the 12 trials, 5 (42%) trials presented a low risk of bias. The mean duration of the interventions was 6.6 (SD 2.8) weeks. Two-thirds of the interventions were based on cognitive behavioral therapy alone or included it in combination with cognitive control therapy, positive psychology, brief behavioral activation, or mindfulness- and acceptance-based therapy. With no evidence of publication bias, a pooled analysis of 83% (10/12) of the trials and 86% (12/14) of the interventions showed that app-based interventions, compared with a control group receiving usual care or minimal intervention, produced a moderate reduction in depressive symptoms (standardized mean difference [SMD] −0.51, 95% CI −0.69 to −0.33; 2018/2859, 70.58% of the participants; *I*^2^=70%). Our meta-regression analyses indicated that there was a greater reduction in symptoms of depression (*P*=.04) in trials that included participants with moderate to severe depression (SMD −0.67, 95% CI −0.79 to −0.55), compared with trials with participants exhibiting mild to moderate depression (SMD −0.15, 95% CI −0.43 to −0.12).

**Conclusions:**

App-based interventions targeted at people with depression produce moderate reductions in the symptoms of depression. More methodologically robust trials are needed to confirm our findings, determine which intervention features are associated with greater improvements, and identify those populations most likely to benefit from this type of intervention.

**Trial Registration:**

PROSPERO CRD42019145689; https://www.crd.york.ac.uk/prospero/display_record.php?RecordID=145689

## Introduction

### Background

Worldwide, approximately 350 million people are affected by depression [1]. In 2010, it was estimated to be the second largest contributor to the global disease burden [2], and by 2030, it is expected to become the leading contributor [3]. Depression is a highly prevalent condition that affects approximately 4.4% of the world’s population [4]. It can have a negative impact on one’s mood and cause emotional distress, potentially interfering with daily functioning [5]. Symptoms of depression range in severity (mild to severe) and duration (months to years) [6]. Depression is the leading contributor to suicide, accounting for approximately 800,000 deaths per year [7]. There is an increasing number of people living with depression worldwide, especially in low-income countries [8,9]. Even in high-income countries, most patients with depression do not receive treatment [10].

The digital market is full of apps designed to improve the mental health of people with depression; however, most of them remain untested in clinical trials and suffer from numerous limitations, such as being designed without content based on the recommendations of experts [11,12]. Therefore, there is a potential risk in the use of such apps, as their therapeutic benefits have not been proven. A recent review of apps targeting depression and anxiety-related conditions [13] observed that the techniques used by some apps were not based on evidence, and in some cases, the manifestation of the techniques promoted by apps could be potentially harmful.

Despite the proliferation of systematic reviews examining the impact of mobile health (mHealth) interventions on mental health during the last decade, the available base of evidence concerning the impact of mobile apps for treating people with depression is still weak. Most of the available reviews offer an overview of the impact of mental mHealth interventions but do not focus on their specific impact on depression [14-18]. A small portion of reviews specifically examine the impact of mHealth interventions on depression; however, some of them rely on user evaluations rather than on evidence from trials [13,17,19]. Although a recent review [20] examined the impact of mHealth interventions on depression, most of the included interventions targeted other mental health problems (insomnia, bipolar disorder, anxiety, and amnesia, among others). To the best of our knowledge, no previous systematic review of randomized controlled trials (RCTs) has evaluated the impact of mHealth interventions specifically designed to improve depressive symptoms in people with depression.

Notwithstanding the above, the use of mHealth technologies for the treatment of symptoms of depression remains very attractive, as such technologies could offer potential benefits in terms of patient autonomy, the prevention of relapse, and lowering costs [14,20]. Community health representatives perceive mHealth technologies as adequate tools for actively involving patients in the management of chronic diseases [21]. Apart from intrinsic barriers to treatment, such as availability, affordability, and time constraints, people’s attitudes also play an important role in non–treatment-seeking behavior [22]. Several barriers that limit the acceptability and adherence to traditional, face-to-face psychotherapy have been described, including the low self-perceived need for treatment, low mental health literacy, high self-reliance, and fear of stigmatization [22,23]. App-based psychological interventions are attractive because of their potential to overcome these barriers.

### Objectives

The aim of this systematic review is to analyze the impact of app-based psychological interventions designed to reduce depressive symptoms in people with depression.

## Methods

### Overview

We conducted a systematic literature review following the Cochrane recommendations [24]. We followed the PRISMA (Preferred Reporting Items for Systematic Reviews and Meta-Analyses) guidelines for planning, conducting, and reporting this study [25]. The review protocol was registered with PROSPERO (CRD42019145689).

### Data Sources and Searches

We designed specific search strategies for biomedical and behavioral science databases (MEDLINE, Embase, PsycInfo, CINAHL, Web of Science, and Cochrane Central Register of Controlled Trials) and combined Medical Subject Headings terms and free-text keywords (Multimedia Appendix 1). We searched the databases from inception to December 23, 2020. We used EndNote X8 to create a bibliographical database and Rayyan to screen relevant records [26].

### Inclusion and Exclusion Criteria

We included empirical studies examining the impact of app-based psychological interventions delivered through smartphones and aimed at reducing depressive symptoms in people with depression compared with a nonactive control group (ie, treatment as usual, waiting-list control, or where minimal intervention was used to ensure blinding or masking). In terms of participants, we included studies involving participants with depressive symptoms of all ages and education levels as assessed using a structured clinical interview conducted according to internationally recognized standards (eg, the International Statistical Classification of Diseases and Related Health Problems and the Diagnostic and Statistical Manual of Mental Disorders) or the presence of significant depressive symptoms established using a validated screening measure (eg, the Patient Health Questionnaire and the Beck Depression Inventory). In terms of the intervention, we included studies that evaluated psychological interventions delivered through an app aimed at reducing depressive symptoms. Although multifaceted interventions were considered, to be included the app needed to have been the main component of the interventions, which were included regardless of the therapeutic orientation upon which they were based. In terms of outcomes, we included studies examining the impact of the intervention on depression severity, as measured using structured clinical interviews or validated screening measures. We included RCTs that were individually randomized and cluster randomized. We included studies in English and Spanish. Letters were excluded from the editor, editorials, study protocols, and conference abstracts. We excluded studies with intervention periods <2 weeks (as we consider this to be the minimum time necessary for changes in depressive symptoms to occur) and those with <50 randomized participants (to minimize the risk of bias arising from small sample sizes [27]).

### Study Selection

In all, 2 of the 4 reviewers (MJSR, MAFD, RZC, and AC) screened all titles and abstracts for potentially eligible papers and subsequently assessed full-text papers against the eligibility criteria. They were blinded to each other’s decisions. All disagreements were resolved by reaching a consensus or by involving a third reviewer.

### Data Extraction and Quality Assessment

In all, 2 of the 4 reviewers (MJSR, MAFD, RZC, and AC) independently extracted quantitative data with respect to the outcomes and characteristics of the studies and interventions in the included papers. Information was extracted and entered into a standardized Microsoft Excel spreadsheet. Discrepancies among the data extractors were discussed until a consensus was reached. We contacted the authors of the included papers to request additional data when needed.

We extracted information concerning the characteristics of the trials (study design, sample size, country, setting, participants, and type of comparator), intervention (length, frequency of use, and psychological theories or techniques used), and outcomes (changes in overall depression). In all, 2 of the 4 reviewers (MJSR, MAFD, RZC, and AC) independently assessed the risk of bias in the studies selected for the meta-analyses using the Cochrane Risk of Bias tool [28]. Discrepancies were discussed among peers to reach a consensus.

### Data Synthesis and Analysis

We conducted a narrative and tabulated synthesis of the findings of the included studies. We pooled data to summarize the progress made in depressive symptoms throughout the intervention and compared interventions to their relevant comparator groups. We anticipated that the included trials would vary in their settings, methods, and designs. Therefore, we used a random effects model to pool the data. Patient-reported measures for depression vary from trial to trial; therefore, we used the Cohen method to calculate pooled effect sizes based on standardized mean differences (SMDs). When needed, we reversed the scale scores (by multiplying them by −1), so that higher scores consistently conveyed higher levels of depression at all scales.

When the SD of the change between baseline and postintervention levels was not reported for either the intervention or the control group, we derived them from baseline and final SDs, assuming a degree of correlation of 0.5. Heterogeneity was quantified using the *I*^2^ statistic, and *I*^2^>50% was considered evidence of substantial heterogeneity. The sources of heterogeneity were explored using the Galbraith plots. Publication bias was examined using funnel plots, and the presence of asymmetry was assessed using the Begg [29] and Egger [30] tests. Meta-analyses were conducted with STATA (version 12.0; StataCorp), using the command *metan*. We conducted a range of exploratory post hoc subgroup and bivariate meta-regression analyses to explore the factors that may affect the effectiveness of smartphone interventions. On the basis of the available evidence, we decided to analyze the following potential moderators: participants’ depression severity (mild to moderate vs moderate to severe) [31,32], therapeutic approaches (cognitive behavioral therapy [CBT] vs CBT plus other approaches vs behavioral activation) [20], intervention duration (1-7 vs 8-12 weeks) [31], comparator (usual care vs minimal intervention) [15,32], components of the intervention (multifaceted vs single-component interventions) [31], communication directionality (unidirectional vs bidirectional communication) [20], and the method used to assess depression (diagnostic instrument vs validated self-reported measure).

### Transparency

The lead author affirms that the manuscript is an honest, accurate, and transparent account of the study being reported; that no important aspects of the study have been omitted; and that any discrepancies from the study as it was planned have been explained (and, if relevant, reported)**.**

## Results

### Search Results

**Figure 1:**
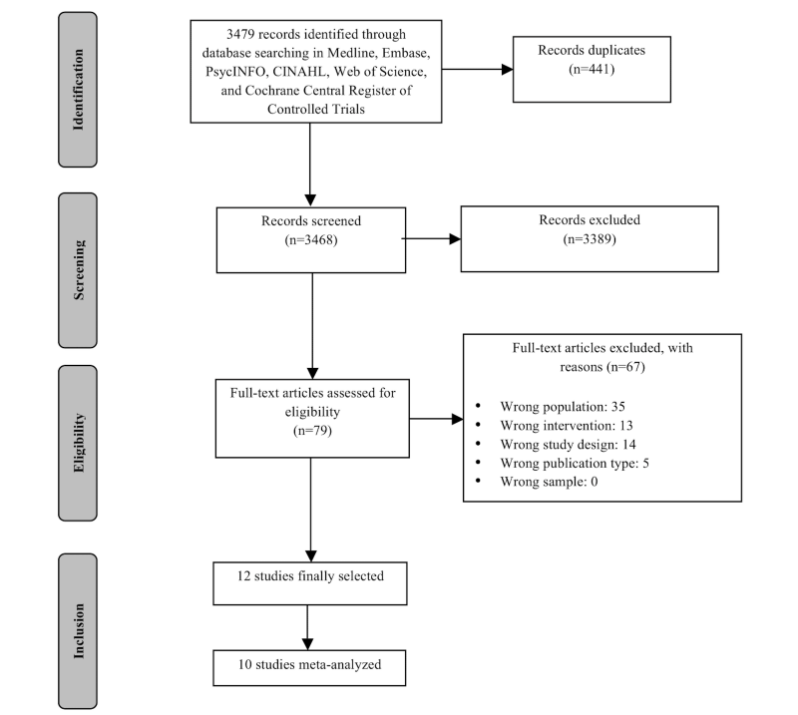
PRISMA (Preferred Reporting Item for Systematic Reviews and Meta-Analyses) flow diagram.

Our search results are summarized in the following PRISMA (Preferred Reporting Items for Systematic Reviews and Meta-Analyses) flow diagram (Figure 1). Our initial search identified a total of 3468 unique citations. Screening the titles and abstracts of these studies resulted in the inclusion of 79 citations for further review. After full-text reviews, 12 trials evaluating 14 different interventions were included in the present systematic review [33-44].

Of these, 83% (10/12) of the trials were included in the meta-analysis and 17% (2/12) of the trials were excluded from the meta-analysis owing to a lack of available data.

### Characteristics of the Included Studies and Interventions

A detailed description of the characteristics of the included trials is provided in Table 1 and in Multimedia Appendix 2 [33-44]. All trials consisted of individually RCTs. In all, 25 % (3/12) of the trials included people with mild to moderate depression [34,39,41], 17% (2/12) of the trials included people with mild to severe depression [33,40], 42% (5/12) of the trials included people with moderate to severe depression [35,36,42-44], 8% (1/12) of the trials included people with a diagnosis of major depression [38], and 8% (1/12) of the trials included people with a self-reported need for help with their depressive symptoms [37]. The total combined sample size for all included trials was 2859 participants. The mean (SD) number of participants per trial was 238 (182), ranging from 52 to 626. In all, 58% (7/12) of the trials were conducted in the United States [33-35,39-41,43], with 8% (1/12) each in Japan [38], Korea [36], Australia [42], Germany [37], and China [44]. A total of 42% (5/12) of the trials took place in a community setting [20,33,39,40,42], 42% (5/12) in hospitals or health organizations [34,36-38,44], and 17% (2/12) in a primary care setting [35,43]. The primary outcome of all included trials was a reduction in depressive symptoms (Table 2). A total of 58% (7/12) of the studies [34,37-39,42-44] compared the intervention against a waiting-list control group, whereas the remaining 42% (5/12) of the trials [33,35,36,40,41] compared the app intervention to another app or a waiting-list control group.

**Table 1:** Characteristics of included trials.

Characteristics		Value
**Year the study was published (N=12), n (%)**		
	2015-2016	4 (33)
	2017-2018	3 (25)
	2019-2020	5 (42)
Number of participants, N; mean (SD; range)		2859; 238 (182; 52-626)
Age of participants (years), mean (SD)		36.12 (10.21)
**Gender of participants (N=2859), n (%)**		
	Male	912 (31.8)
	Female	1899 (66.4)
	Others	48 (1.67)
**Country (N=12), n (%)**		
	United States	7 (58.3)
	Japan	1 (8.3)
	South Korea	1 (8.3)
	Australia	1 (8.3)
	Germany	1 (8.3)
	China	1 (8.3)
**Instrument used to measure depression^a^ (N=12), n (%)**		
	Patient Health Questionnaire-9	6 (50)
	Patient Health Questionnaire-8	3 (25)
	Beck Depression Inventory-II	2 (16.7)
	Depression, Anxiety, Stress Scale-21	1 (8.3)
	Center for Epidemiological Studies-Depression Scale	2 (16.7)
**Setting (N=12), n (%)**		
	Community	5 (41.7)
	Hospital or health organizations	6 (50)
	Primary care	1 (8.3)
**Type of approach or psychotherapy^a^ (N=12), n (%)**		
	Cognitive behavioral therapy	8 (66.7)
	Cognitive control therapy	2 (16.7)
	Brief behavioral activation	1 (8.3)
	Positive psychology	2 (16.7)
	Mindfulness	1 (8.3)
	Acceptance-based therapy	1 (8.3)
Duration of intervention in weeks, mean (SD; range)		6.6 (2.8; 4-12)

^a^Percentages exceeding 100% as categories are not mutually exclusive.

**Table 2:** Summary of findings.

Study	Severity of depression and instrument (cut point)	Intervention A (n) and intervention B (n)	Comparator (n)	Length	Study design	Main results
Arean et al [33]	Mild to severe depression without suicidal ideation PHQ-9^a^ (score >5 or score on item 10 ≥2)	EVO app (N=221) iPST^b^ app (N=209)	Usual care (N=206)	4 weeks	Effectiveness	No significant differences observed between the 2 interventions compared with the control after the intervention and at follow-up. Moderately depressed participants had a greater response to Project: EVO (28/56, 50%) and iPST (39/79, 49%) than the control arm (24/76, 32%; χ2=6.46; *P*=.04) in remission rates.
Birney et al [34]	Mild to moderate depression PHQ-9 (score of 10-19)	MoodHacker (N=150)	Minimal intervention (N=150)	6 weeks	Efficacy	Compared with the control group, the MoodHacker app had significant effects on symptoms of depression in users (*P*=.01; partial η2=0.021) after the intervention period.
Dahne et al [35]	Moderate to severe depression without suicidal ideation PHQ-8^c^ (score >10) and BDI-II^d^ (score >13)	Moodivate app (N=24) Moodkit app (N=19)	Minimal intervention (N=9)	8 weeks	Efficacy	Over time and compared with the control group, participants using either app provided evidence of significant decreases in depressive symptoms that were sustained over the trial period.
Ham et al [36]	Moderate to severe depression BDI-II (score >16)	HARUToday (N=21)	HARUCard (attention control group) (N=21) Waiting list (N=21)	10 weeks	Effectiveness	BDI-II scores of the HARUToday group decreased significantly after the intervention compared with the attention control (HARUCard) and waiting-list control groups (*P*=.01).
Lüdtke et al [37]	Subjective need for help with depressive symptoms PHQ-9 (N/A^e^)	Be Good to Yourself (N=45)	Usual care (waiting list) (N=45)	4 weeks	Efficacy	Depressive symptoms decreased in both groups after the intervention period, without significant differences among groups (*P*=.95).
Mantani et al [38]	Diagnosed major depression PRIME-MD^f^ and BDI-II (score ≥10)	Kokoro-app (N=81)	Usual care (N=83)	9 weeks	Effectiveness	The intervention group improved significantly compared with the control group (95% CI 1.23-3.72; *P*<.001; SMD^g^ 0.40). The benefits were maintained during the follow-up period.
Moberg et al [39]	Mild to moderate depression PHQ-8 (score between 5 and 14)	Pacifica app (N=253)	Usual care (waiting list) (N=247)	4 weeks	Effectiveness	Participants in the intervention group demonstrated significantly greater decreases in depression. The Group x Time interaction effect size is as follows: Cohen d 0.54; *P*<.001. Rates of clinical significance change after the intervention: Pacifica, 42% (33/79); waiting list 17% (17/101); *P*<.001.
Pratap et al [40]	Clinically significant depressive symptoms PHQ-9 (score ≥5 or score on item 10 ≥2)	EVO (N=83) iPST (N=112)	Minimal intervention (daily health tips; N=79)	4 weeks	Efficacy	No significant differences were observed in depression outcomes among the 3 groups.
Roepke et al [41]	Mild to moderate depression CES-D^h^ (score ≥16)	CBT-PPT^i^ SB^j^ (N=93) General SB (N=97)	Usual care (waiting list) (N=93)	4 weeks	Effectiveness	After treatment and during follow-up, General SB participants saw greater reductions in depression scores than the control group (*P*<.001).
Tighe et al [42]	Moderate to severe depression PHQ-9 (score >10)	Ibobbly (N=31)	Usual care (waiting list) (N=30)	6 weeks	Effectiveness	The app group showed statistically significant reductions in depression scores compared with the control group (*P*=.007).
Graham et al [43]	Moderate to severe depression PHQ-8 (score >10)	IntelliCare platform (N=74)	Usual care (waiting list) (N=72)	8 weeks	Effectiveness	IntelliCare participants achieved greater reductions in depression and higher odds of recovery compared with the controls (odds ratio 3.25; 95% CI 1.54-6.86).
Guo et al [44]	Moderate to severe depression CES-D (score ≥16)	Run Love (Wechat platform; N=150)	Usual care (waiting list) (N=150)	12 weeks	Effectiveness	The intervention group saw significantly reduced depression severity compared with the control group (from 23.9 to 17.7 vs from 24.3 to 23.8; mean difference −5.77, 95% CI −7.82 to −3.71; *P*<.001).

^a^PHQ-9: Patient Health Questionnaire-9.

^b^iPST: Problem-solving therapy app.

^c^PHQ-8: Patient Health Questionnaire-9.

^d^BDI-II: Beck Depression Inventory.

^e^N/A: not applicable.

^f^PRIME-MD: Primary Care Evaluation of Mental Disorders.

^g^SMD: standardized mean difference.

^h^CES-D: Center for Epidemiologic Studies-Depression Scale.

^i^CBT-PPT: cognitive behavioral therapy and positive psychotherapy.

^j^SB: SuperBetter.

The mean duration of the interventions was 6.6 (SD 2.8) weeks, with a range of 4-12 weeks. Two-thirds of the interventions were based on CBT alone or CBT in combination with cognitive control therapy, positive psychology, brief behavioral activation, or mindfulness and acceptance-based therapy. There was variability in terms of use recommendations, with participants being recommended daily or almost daily use in 67% (8/12) of the trials [33-37,39-41] and receiving no use recommendations in 33% (4/12) of trials [38,42-44].

### Risk of Bias

**Figure 2:**
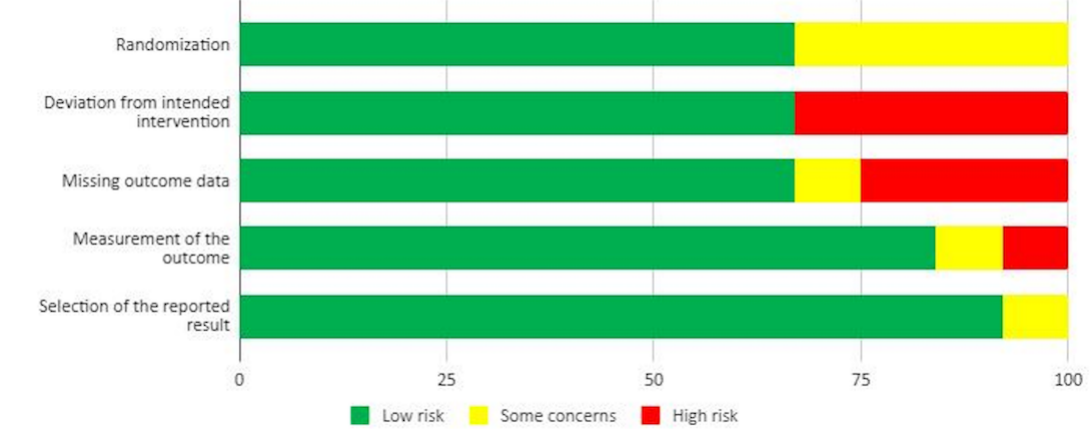
Risk of bias assessment.

The results of the general risk of bias assessment are shown in Figure 2. In all, 42% (5/12) of the included studies showed a low risk of bias, 17% (2/12) of the studies showed a low risk of bias in 4 of the 5 domains considered, and the remaining 42% (5/12) of the studies showed an unclear risk. The most frequent biases included the following domains: deviation from intended intervention (high risk in 4/12, 33% studies), randomization (some concerns in 4/12, 33% studies), missing outcome data (high risk in 3/12, 25% studies and some concerns in 1/12, 8% study), and measurement of the outcome (high risk in 1/12, 8% study and some concerns in 1/12, 8% study). Our assessment of the risk of bias in individual studies is shown in Multimedia Appendix 3 [33-44].

### Impact of the Apps

In terms of impact, in the only trial that included patients with clinically diagnosed major depression [38], the authors reported that a CBT-based app intervention (Kokoro-App) improved depressive symptoms in users when compared with a waiting-list control group. A similar beneficial effect was observed in most (10/14, 71%) of the remaining app interventions: a CBT-based app (MoodHacker) [34] improved symptoms of depression compared with accessing relevant internet sites about depression. An app based on CBT and mindfulness (Pacifica App) significantly reduced depressive symptoms in users when compared with a waiting-list control group [39]. An app called SuperBetter (SB) based on CBT and positive psychotherapy strategies SB and an app that focused on self-esteem and acceptance (General SB) produced greater reductions in depression scores in users than in waiting-list participants [41]. In all, 14% (2/14) of the apps based on brief behavioral activation and CBT (Moodivate and Moodkit, respectively) produced significant decreases in depressive symptoms when compared with usual care [35], whereas another similar app (HARUToday app) [36] was also shown to have significantly reduced depressive symptoms compared with both a minimal intervention and a waiting-list control group. In a remote community setting [42], an app based on acceptance-based therapy (Ibobbly app) significantly reduced symptoms of depression in users compared with waiting-list participants. A platform containing 5 clinically focused CBT– and positive psychology–based apps [43] produced larger reductions in symptoms of depression and higher recovery rates than those seen in waiting-list participants. An app based on cognitive behavioral stress management and automatic progress monitoring (Run4Love app) [44] significantly reduced depression severity in users compared with a waiting-list control group.

Few of the interventions, however, did not consistently demonstrate the intended effect: a trial comparing 2 active interventions (Project EVO, a cognitive control app and iPST, a problem‐solving therapy app) against a minimal intervention control group [33] observed that both apps had a greater effect on mood in users than the control group. However, when the same 2 interventions were subsequently evaluated in a separate trial with a high proportion of Hispanic and Latino participants [40], no significant differences were observed. A trial comparing the effect of the app Be Good to Yourself (based on CBT and mindfulness) in users with a waiting-list control group found that depressive symptoms decreased in both groups, with no significant between-group differences [37].

### App Use

In all 50% (6/12) of the trials reported results concerning the app use levels. Across these studies, the data were reported using a number of different metrics (eg, percentage of participants who completed the intervention activities, number of downloads, and average use time), hindering our attempts to pool it.

App use varied widely across studies: 17% (2/12) of the trials reported that around 80% of their participants used the app as instructed (in a study by Tighe et al [42], 34/40, 85% of the participants completed all the activities and in a study by Graham et al [43], 119/146, 81.5% of the participants had some app use). However, app use was significantly lower in 3 trials: Arean et al [33] reported that 57.9% (243/420) of participants did not download the app, Dahne et al [35] reported that 43% (9/21) of participants used the app the number of times required, and Roepke et al [41] reported that 15% of the participants downloaded the app or used it to the complete content (Multimedia Appendix 3).

App use was associated with higher levels of depression at the baseline [33]. A dose-response effect was examined in 17% (2/12) of the studies: in Moberg et al [39], no significant association between overall app engagement (defined as the total number of log-ins) and symptom improvement was observed, whereas in Roepke et al [41], participants who actually downloaded General SB or the complete CBT and positive psychotherapy content achieved a significantly greater decrease in depressive symptoms.

### The Pooled Effects of Smartphone Interventions for Reducing Depressive Symptoms

**Figure 3:**
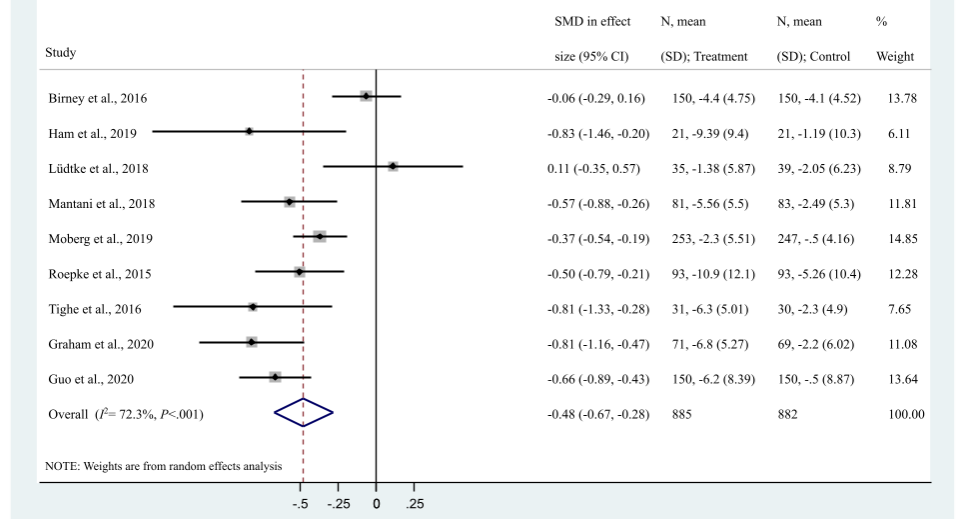
Effect of apps on depressive symptoms compared with active treatment and control conditions. CBT-PPT: cognitive behavioral therapy and positive psychotherapy; SB: SuperBetter; SMD: standardized mean difference.

We pooled data from 10 trials that assessed 12 interventions (Figure 3) [34-39,41-44]. Data from the remaining 17% (2/12) of the trials included in our review were not available despite our attempts to contact the authors. According to a random effects meta-analysis, the interventions had a statistically significant and moderate effect in reducing depressive symptoms compared with control conditions in which participants received usual care or minimal intervention (SMD −0.51, 95% CI −0.69 to −0.33; 2018/2859, 70.58% of the participants; *P*<.001; *I*^2^=70%). In a sensitivity analysis that excluded from the meta-analysis, the 2 trials that most contributed to the high levels of observed heterogeneity (ie, Dahne et al [35] and Lüdtke et al [37]), the pooled impact of the interventions was greater (SMD −0.61, 95% CI −0.74 to −0.48; 1644/2859, 57.5% of the participants; *P*<.001; *I*^2^=34%). Begg and Egger tests suggested the absence of publication bias in both meta-analyses (*P*=.53 and *P*=.89, respectively, for the main meta-analysis; and *P*=.31 and *P*=.93, respectively). In a second sensitivity analysis excluding 33% (4/12) of the trials with high risk of bias, a moderate statistically significant effect was still observed (SMD −0.41, 95% CI −0.71 to −0.10; 781/2859, 27.31% of the participants; *P*=.009; *I*^2^=71.6%), with the absence of publication bias according to Egger test (*P*=.53) and Begg test (*P*=.88).

According to our post hoc subgroup analyses (Table 3), the interventions led to better results in trials focusing on moderate to severe depression symptomatology (6/12, 50% of the trials and 9/14, 64% of the interventions [36,38,41-44]; SMD −0.67; 95% CI −0.79 to −0.55; 1144/2859, 40.01% of the participants; *I*^2^=0.0%) compared with trials involving patients with mild to moderate symptoms of depression (3/12, 25% of the trials and 4/14, 28% of the interventions [34,37,39]; SMD −0.15, 95% CI −0.43 to 0.12; 874/2859, 30.59% of the participants; *I*^2^=69.3%). This subgroup difference was statistically significant according to our meta-regression analysis (*P*=.003). The effects of interventions versus usual care (SMD −0.58, 95% CI −0.76 to −0.40) were greater than the effects of interventions versus an active control group receiving minimal intervention (SMD 0.11, 95% CI: −0.32 to 0.10). However, this difference was not statistically significant according to meta-regression (*P=*.076). The differences among the remaining subgroups were smaller and not statistically significant.

**Table 3:** Subgroup post hoc analyses.

Characteristics		Interventions (n=14), n (%)	Sample size (smartphone/control)	Meta-analysis, SDM^a^ (95% CI)	Heterogeneity		Between-group tests, coefficient (95% CI)	
					*I*^2^ (%)	*P* value		
**Depression severity**								−0.49 (−0.76 to −0.22)
	Mild to moderate	3 (21)	438/436	−0.16 (−0.43 to 0.12)	69.3	.04		
	Moderate to severe	9 (64)	587/557	−0.67 (−0.79 to −0.55)	0.0	.68		
**Type of psychotherapy**								0.08 (−0.14 to 0.30)
	CBT^b^	4 (28)	271/263	−0.63 (−0.81 to −0.46)	0.0	.85		
	CBT + positive psychology	4 (28)	411/405	−0.55 (−0.95 to −0.15)	87.1	<.001		
	CBT + mindfulness	2 (14)	288/286	−0.18 (−0.64 to 0.28)	72.7	.06		
	Behavioral activation	2 (14)	55/39	−0.65 (−1.09 to −0.21)	2.2	.31		
**Intervention duration**								−0.24 (−0.64 to 0.16)
	1-7 weeks	6 (43)	659/652	−0.41 (−0.68 to −0.14)	80.5	<.001		
	8-12 weeks	6 (43)	366/341	−0.66 (−0.81 To −0.50)	0.0	.80		
**Comparator**								−0.41 (−0.87 to 0.05)
	Usual care	9 (64)	832/825	−0.58 (−0.76 to −0.40)	62.9	.001		
	Active control (minimal intervention)	3 (21)	193/168	−0.11 (−0.32 to 0.10)	0.0	.56		
**Intervention components**								−0.07 (−0.52 to 0.37)
	Unifaceted	7 (50)	552/520	−0.48 (−0.72 to −0.24)	63.2	.01		
	Multifaceted	5 (35)	473/473	−0.56 (−0.87 to −0.24)	80.3	<.001		
**Directionality**								−0.29 (−0.70 to 0.11)
	Unidirectional communication	8 (57)	702/670	−0.41 (−0.64 to −0.17)	72.8	<.001		
	Bidirectional communication	4 (28)	323/323	−0.68 (−0.84 to −0.52)	0.0	.72		
**Method for assessing depression**								0.07 (−0.68 to 0.81)
	Diagnostic instrument	1 (7)	81/83	−0.57 (−0.88 to −0.26)	N/A^c^	N/A		
	Validated self-reported measure	11 (78)	944/911	−0.50 (−0.70 to −0.30)	72.4	<.001		

^a^SMD: standardized mean difference.

^b^CBT: cognitive behavioral therapy.

^c^N/A: not applicable (subgroup with only 1 study).

## Discussion

### Principal Findings

In this systematic review and meta-analysis, we identified 12 RCTs examining the impact of 14 smartphone apps specifically designed to reduce depressive symptoms in people with depression. We observed that 71% (10/14) of the interventions led to a significant reduction in depressive symptoms. Our pooled analyses suggest that they had a moderate effect, which was significantly larger in interventions targeted to patients with more severe depression.

### Comparison of the Main Findings With Previous Reviews

All the studies identified in our review have been published within the last 5 years, which underscores the increasing interest in this type of intervention. However, despite a growing number of studies, the available evidence base is limited by the methodological quality of the trials that have been conducted to date, most of which suffer from moderate or substantial risk of bias. This finding is in line with the results of another recent review that concluded that there is still not enough evidence to support the prescription of independent mHealth tools for depression as an adjunctive treatment [13]. Indeed, the difference between the high volume of commercially available apps and the low number of tested, evidence-based apps is striking.

The significant effects observed in our systematic review generally support the findings of previous, broader reviews [14,16,20]. However, in our review, which for the first time, meta-analyzed interventions specifically designed to reduce depressive symptoms, the observed effect size (0.51) was larger than in previous meta-analyses (ranging from 0.33 to 0.38) [14,16,20]. This may be explained by the fact that, contrary to previous meta-analyses, we only included trials comparing the intervention with a control group that received usual care or minimal intervention. Our findings also support the results of a recent systematic review of smartphone apps for depression, which included both observational and experimental studies [45] and observed a decline in depressive symptoms in all the included studies. They additionally collected information on the attitudes of health care professionals, observing that, although they are open to therapeutic app use, professionals have limited knowledge and experience in this field.

Regarding the target population, we observed larger effects in interventions targeting people with moderate to severe depression, whereas in the review by Firth et al [20], the authors observed that mobile apps only reduced depressive symptoms in people with mild to moderate symptoms, with no differences observed in people with major depression. This difference between our review and the review by Firth et al [20] may be partially explained by the larger number of interventions we identified that were targeted to people with severe symptoms of depression (9 trials vs the 2 trials included in the review by Firth et al [20]). In their review of interventions for a broad range of mental conditions, Weisel et al [16] found that app interventions had a significant effect compared with controls for general depression but only when the comparator was a control group receiving usual care.

A recent clinical trial conducted by our team found that a psychoeducational intervention delivered through an app produced significant improvements in the mental health of health care workers on the frontline of the COVID-19 pandemic who were receiving psychotherapy or taking psychotropic drugs [46,47]. Mobile apps present numerous unique advantages, including increased accessibility to the intervention (ubiquitous access) and the fact that they provide access to people who do not seek help for their mental health problems. Thus, mHealth interventions could address the main barriers to help-seeking behaviors, such as geographic location and the stigma associated with mental illness [22,48]. Apps also provide opportunities for users to access the intervention several times a day and when it is most needed [12]. Considering their potential to improve access to mental health services and as many people do not feel the need for treatment [22], apps may be able to motivate users to seek a diagnosis or treatment, as evidenced by an app for the evaluation of depression. In this sense, participants in 92% (11/12) of the studies in the present review were encouraged to use the mobile app several times a week to daily, in some cases stimulating use with reminders. However, the available data suggest that app use is generally low (around 80% of the participants used the app as instructed in 2/12, 17% of the studies, whereas in 4/12, 33% of the studies use was <50%) concerning app use suggested from the data provided, it can be inferred that there have been few downloads of the apps, that those who downloaded them, the use has been limited and that a greater number of apps does not translate into significant improvements in depressive symptoms.

It seems that the emerging use of apps to take care of people’s mental health is unstoppable, whether it is partially or in combination with the intervention of a therapist [16,17]. The evidence from the present review and meta-analysis suggests that interventions delivered via smartphones have a beneficial effect on depressive symptoms. Understanding which psychological interventions delivered through smartphones are the best and what types of patients they can best serve will require more research. Embedding process evaluations in future RCTs would provide information on mechanisms of action and a better understanding of the contexts and premises under which mHealth interventions produce beneficial effects.

New technologies are increasingly present in our lives, and mental health is not an exception. As more mental health apps are created, we will need to focus on tailoring them to more personalized populations and users so that they are likely to be more effective. Future studies should explore reliable frameworks for making use of mental health apps in the context of psychological and psychiatric care.

### Strengths and Limitations of the Review

To the best of our knowledge, this is the first systematic review and meta-analysis to examine the impact of mHealth interventions specifically designed for people with depression. The strengths of this review are the large number of bibliographic databases searched, the fact that study eligibility, data extraction, and risk of bias assessments were conducted by independent senior reviewers, and the statistical analyses adhered to best-practice recommendations [24]. The current systematic review is not without limitations. Our bibliographic searches were restricted to publications in English and Spanish. In addition, we did not search for unpublished data. Both aspects may have hindered our ability to identify additional relevant trials. The differences in severity of depression, the time of the treatment received, and the differences among the studies made it difficult to establish the most effective individual components (active ingredients) of the included interventions. These differences are also likely to have contributed to the substantial heterogeneity observed in the meta-analysis (*I*^2^=70%). However, the heterogeneity was reduced to 34% in a sensitivity analysis, excluding the 17% (2/12) of the trials that most contributed to this high level of heterogeneity. The results of the sensitivity analysis support the finding that these interventions have a moderate effect. The use of medication in addition to psychological treatment may also influence treatment outcomes, but we were not able to explore this in the review. Finally, we acknowledge the following two deviations from our published protocol: (1) the inclusion of studies that assessed depression using self-reported tools rather than diagnostic instruments (as we only identified 1 trial using a diagnostic instrument), and (2) the exclusion of studies with intervention periods <2 weeks or with <50 participants.

### Conclusions

mHealth interventions targeted at people with symptoms of depression produce moderate reductions in these symptoms, with larger effects being seen in people with more severe symptoms. Although the available evidence seems to follow this line, there is still insufficient evidence to support the prescription of mHealth tools to improve depressive symptoms or as an adjunct treatment. Future research should focus on conducting more clinical trials with solid methodological foundations to investigate the impact of digital psychological interventions for the treatment of depression.

## Appendix

Multimedia Appendix 1 Search strategy.

Multimedia Appendix 2 Characteristics of the study interventions.

Multimedia Appendix 3 Quality assessment of included studies.

## Abbreviations

CBT: cognitive behavioral therapy
mHealth: mobile health
RCT: randomized controlled trial
SB: SuperBetter
SMD: standardized mean difference
