# Supplementary material for: Impact of Smartphone App–Based Psychological Interventions for Reducing Depressive Symptoms in People With Depression: Systematic Literature Review and Meta-analysis of Randomized Controlled Trials
Source: JMIR Mhealth Uhealth. 2022 Jan 27;10(1):e29621. doi: 10.2196/29621 (PMC8832272; doi:10.2196/29621)
Supplement: Multimedia Appendix 1 [file mhealth_v10i1e29621_app1.docx]

**Multimedia Appendix 1. Search strategy**

EMBASE: 23 December 2020

|  | Searches |
| --- | --- |
| 1 | depression'/exp |
| 2 | 'depress*':ab,ti |
| 3 | bipolar':ab,ti |
| 4 | automutilation'/exp |
| 5 | 'self-harm':ab,ti |
| 6 | 'affective psychosis'/exp |
| 7 | 'affective disorder*':ab,ti |
| 8 | 'mood disorder*':ab,ti |
| 9 | 'depression'/exp OR 'depress*':ab,ti OR 'bipolar':ab,ti OR 'automutilation'/exp OR 'self-harm':ab,ti OR 'affective psychosis'/exp OR 'affective disorder*':ab,ti OR 'mood disorder*':ab,ti |
| 10 | 'mhealth'/exp |
| 11 | 'mhealth':ab,ti |
| 12 | 'm-health':ab,ti |
| 13 | 'mobile health':ab,ti |
| 14 | 'mobile device':ab,ti |
| 15 | 'mobile application'/exp |
| 16 | ('mobile' OR 'smart' OR 'tablet') NEAR/5 (app* OR application* OR phone*) |
| 17 | 'smartphone':ab,ti |
| 18 | 'mobile phone':ab,ti |
| 19 | 'mobile phone'/exp |
| 20 | 'cell phone':ab,ti |
| 21 | 'mhealth'/exp OR 'mhealth':ab,ti OR 'm-health':ab,ti OR 'mobile health':ab,ti OR 'mobile device':ab,ti OR 'mobile application'/exp OR ('mobile' OR 'smart' OR 'tablet') NEAR/5 (app* OR application* OR phone*) OR 'smartphone':ab,ti OR 'mobile phone':ab,ti OR 'mobile phone'/exp OR 'cell phone':ab,ti |
| 22 | 'crossover procedure':de OR 'double-blind procedure':de OR 'randomized controlled trial':de OR 'single-blind procedure':de OR random*:de,ab,ti OR factorial*:de,ab,ti OR crossover*:de,ab,ti OR ((cross NEXT/1 over*):de,ab,ti) OR placebo*:de,ab,ti OR ((doubl* NEAR/1 blind*):de,ab,ti) OR ((singl* NEAR/1 blind*):de,ab,ti) OR assign*:de,ab,ti OR allocat*:de,ab,ti OR volunteer*:de,ab,ti |
| 23 | ('depression'/exp OR 'depress*':ab,ti OR 'bipolar':ab,ti OR 'automutilation'/exp OR 'self-harm':ab,ti OR 'affective psychosis'/exp OR 'affective disorder*':ab,ti OR 'mood disorder*':ab,ti) AND ('mhealth'/exp OR 'mhealth':ab,ti OR 'm-health':ab,ti OR 'mobile health':ab,ti OR 'mobile device':ab,ti OR 'mobile application'/exp OR ('mobile' OR 'smart' OR 'tablet') NEAR/5 (app* OR application* OR phone*) OR 'smartphone':ab,ti OR 'mobile phone':ab,ti OR 'mobile phone'/exp OR 'cell phone':ab,ti) AND ('crossover procedure':de OR 'double-blind procedure':de OR 'randomized controlled trial':de OR 'single-blind procedure':de OR random*:de,ab,ti OR factorial*:de,ab,ti OR crossover*:de,ab,ti OR ((cross NEXT/1 over*):de,ab,ti) OR placebo*:de,ab,ti OR ((doubl* NEAR/1 blind*):de,ab,ti) OR ((singl* NEAR/1 blind*):de,ab,ti) OR assign*:de,ab,ti OR allocat*:de,ab,ti OR volunteer*:de,ab,ti) |
| 24 | (('depression'/exp OR 'depress*':ab,ti OR 'bipolar':ab,ti OR 'automutilation'/exp OR 'self-harm':ab,ti OR 'affective psychosis'/exp OR 'affective disorder*':ab,ti OR 'mood disorder*':ab,ti) AND ('mhealth'/exp OR 'mhealth':ab,ti OR 'm-health':ab,ti OR 'mobile health':ab,ti OR 'mobile device':ab,ti OR 'mobile application'/exp OR ('mobile' OR 'smart' OR 'tablet') NEAR/5 (app* OR application* OR phone*) OR 'smartphone':ab,ti OR 'mobile phone':ab,ti OR 'mobile phone'/exp OR 'cell phone':ab,ti) AND ('crossover procedure':de OR 'double-blind procedure':de OR 'randomized controlled trial':de OR 'single-blind procedure':de OR random*:de,ab,ti OR factorial*:de,ab,ti OR crossover*:de,ab,ti OR ((cross NEXT/1 over*):de,ab,ti) OR placebo*:de,ab,ti OR ((doubl* NEAR/1 blind*):de,ab,ti) OR ((singl* NEAR/1 blind*):de,ab,ti) OR assign*:de,ab,ti OR allocat*:de,ab,ti OR volunteer*:de,ab,ti)) AND (2006:py OR 2007:py OR 2008:py OR 2009:py OR 2010:py OR 2011:py OR 2012:py OR 2013:py OR 2014:py OR 2015:py OR 2016:py OR 2017:py OR 2018:py OR 2019:py OR 2020:py) AND [embase]/lim NOT ([embase]/lim AND [medline]/lim) |
