# Supplementary material for: Impact of Smartphone App–Based Psychological Interventions for Reducing Depressive Symptoms in People With Depression: Systematic Literature Review and Meta-analysis of Randomized Controlled Trials
Source: JMIR Mhealth Uhealth. 2022 Jan 27;10(1):e29621. doi: 10.2196/29621 (PMC8832272; doi:10.2196/29621)
Supplement: Multimedia Appendix 2 [file mhealth_v10i1e29621_app2.docx]

**Multimedia Appendix 2. Characteristics of the study interventions.**

| **Author/**  **Year** | **Setting /**  **Total Sample/**  **Country** | **Intervention/**  **Duration /**  **Recommendation of use /**  **Type of approach or psychotherapy /**  **App usage** | **Evaluation time points (from recruitment)** |
| --- | --- | --- | --- |
| Arean 2016[33] | Community  N=626  USA | (A) EVO App:  Cognitive training by a multitasking game that targets perceptual discrimination abilities, selective attention, and visual and motor tracking skills  4 weeks  6 times a week and 30 minutes per day  Cognitive Control Therapy  (B) Problem Solving Therapy App (iPST):  Focuses on a 7-step model to manage mood  4 weeks  NR  NA  Among the 420 participants, 243 (57.9%) did not download their assign app. Those who used their app at least once used it on average 10.78 (SD 11.44) times.  Higher baseline depression (PHQ-9) was associated with less use (*P*<0.05). | Weekly during all the intervention (4 weeks), 8 and 12 weeks |
| Birney 2016[34] | Mental health organizations  N=300  USA | MoodHacker:  Cognitive and behavioral training through 4 modules (daily mood and activity monitoring; increased engagement in positive behavioral activities; decreased negative thinking and increased positive thinking, and increased practice of gratitude, mindfulness, and strength-based cognitions and behaviors).  6 weeks  Daily use stimulated by daily mails  CBT and Positive Psychology  NA | 6 and 10 weeks |
| Dahne 2018[35] | Primary Care  N=52  USA | (A) Moodivate App:  Includes psychoeducation, identification of life areas, values, and associated activities, daily monitoring and activity planning, mood monitoring, and social support to facilitate completion of difficult activities  8 weeks  At least once per day  Brief behavioral activation  (B) Moodkit App:  Cognitive behavioural App Via the thought-checker feature, users identify maladaptive thoughts and alternative, more rationale thoughts. Users track daily mood while using the app and can view a graph of change in mood over time. The app’s journal function allows users to write responses variety of templates. Via the activity scheduler, users can select from +200 suggested activities or create a new custom activity  8 weeks  At least once per day  CBT  All participants used the App at least once during  the trial, and 42.9% of participants used the app  more than 56 times (i.e., at least once per day on  average). Participants on average had 46.76 (30.10)  App sessions throughout the 8-week trial duration,  spent 3.50 (2.76) minutes using the app per session,  and spent 120.76 (101.02) minutes using the app in  total throughout the trial. | Weekly during all the intervention (8 weeks) |
| Ham 2019[36] | Hospital, cancer centers, portal sites for cancer patients  N=63  Korea | (A) HARUToday:  includes five modules: (1) psychoeducation, (2) behavioral activation, (3) relaxation training, (4) cognitive restructuring, and (5) problem-solving. It is composed of 48 sessions, each of which takes approximately 10–15 minutes to complete. All sessions are composed of four phases: (1) Mood rating, (2) Lesson, (3) Summary, and (4) Quizzes.  10 weeks  One session per day for a total of 10 weeks (66 days) at home, excluding weekends  CBT and Behavioral Activation  (B) HARUCard(attention control group)^$^:  Minimal intervention based on the provision of information related to (1) depression and anxiety, (2) exercise tips, (3) hobbies and travel, (4) movies and books, (5) famous quotes, and (6) artworks  10 weeks  Daily  NA  NA | 12 weeks (2 weeks after completing the intervention) |
| Lüdtke 2018[37] | Outpatient clinic and online forumsthat offer a place for people with depression.  N=90  Germany | Be Good to Yourself  The application consists of 40 self-help strategies and exercises. These exercises are based on CBT and its third wave. Exercises are assigned to four categories: cognitive strategies, mindfulness-based exercises, social-competence skills, and activating exercises. Each exercise is described in less than 150 words and is easily read and performed in a couple of minutes. Each exercise includes a short psychoeducational section as well as instructions for an exercise  4 weeks  Several times a week or more  CBT and Mindfulness  NA | 4 weeks |
| Mantani 2017[38] | Psychiatric clinics and hospitals  N=164  Japan | Kokoro-App:  Eight sessions, including one welcome session, two sessions on self-monitoring, two sessions on behavioral activation, two sessions on cognitive restructuring, and an epilog focusing on relapse prevention. In each session, an explanation of the principles and skills of CBT is provided in the format of instant messenger exchanges among cartoon characters. Included sessions on self-monitoring, behavioral activation, and cognitive restructuring presented by cartoon characters  9 weeks  1 session per week (9 weeks of intervention)  CBT  NA | 5, 9 and 17 weeks |
| Moberg 2019[39] | Community  N=500  USA | Pacifica App:  Guided self-help tool for the management of stress, anxiety, and depression. At onboarding, users select goals on which to work from a list of 8 options. There are also 35 days of Guided Paths that are approximately 10-min audio psychoeducational lessons with paired activities  4 weeks  The app prompts users once per day to rate their mood and, based on their mood rating, recommends activities to improve their mood via Suggested Activities  CBT and Mindfulness  The median number of logins in the App during the 30-day intervention was 19 (range = 1 to 286). No significant association between overall engagement with the App (defined by total number of logins) and symptom improvement was observed. | 4 and 12 weeks |
| Pratap 2018[40] | Community  N=274  USA | (A) EVO App:  Cognitive training by a multitasking game that targets perceptual discrimination abilities, selective attention, and visual and motor tracking  4 weeks  Daily reminders to use the app were sent once a day  Cognitive control therapy  (B) Problem Solving Therapy App (iPST):  Participants are asked to identify an area where they are experiencing problems (e.g. stress, finances, work, etc). Upon selecting an area, more specific problems are provided (for example, depression, anxiety, or anger are listed under the stress domain) and participants are asked to choose a goal to help overcome their problem or are given the option to write in their own goal. From there, participants are asked to identify three strategies to help them achieve their goal (or to write in their strategy)  4 weeks  iPST is meant to be played daily, with the approximate time to solve a problem being 10 minutes  Problem-solving Therapy  NA | Weekly during all the intervention (4 weeks) |
| Roepke 2015[41] | Community  N=283  USA | (A) CBT-PPT Super Better:  5 MODULES – distributed in 2 sets of activities. Uses game mechanics to increase users’ drive to accomplish challenging goals and to build social support through online discussion forums and Facebook integration. A version of SB specially designed for depression, using principles from CBT and PPT pleasure and mastery  4 weeks  At least 10 minutes per day  CBT and Positive Psychotherapy  Of the 93 participants assigned to CBT-PPT SB, 31 participants (33%) downloaded the all the content as intended.  (B) General SuperBetter (SB):  focused on self-esteem and acceptance of the present. For instance, participants were asked to ‘‘practice being present’’ (notice surroundings, breathe deeply, etc.), collect a list of ‘‘awesome qualities’’ others attribute to them, or find a piece of art or music that reminds them to accept life’s ups and downs  4 weeks  At least 10 minutes per day  Third-wave CBT (acceptance-based).  Of the 190 participants assigned to SB, 76% logged in at least once. Number of log-ins ranged from 1 to 274 (mean(SD)= 22(34))  In comparison with WL participants who did not use SB at all (79/ 93), participants who actually downloaded General SB or the complete CBT-PPT content achieved significantly greater decreases in depression. In contrast, those who downloaded PPT only did not fare better than participants who did not use SB at all (in 14 cases it was uncertain whether a WL participant had downloaded SB content before or after the WL period had elapsed. These individuals were excluded here in order to make this a clean treatment-on-treated analysis). | 2, 4 and 6 weeks |
| Tighe 2017[42] | Remote and very remote communities  N=61  Australia | Ibobbly:  3 content modules and 3 self-assessments. Participants were expected to progress unprompted through the content. All activities were required to be completed in sequence. Repetition of activities was encouraged to improve learning and impact  6 weeks  No recommendation  Acceptance-based Therapy  Usage data were available from 40 out of 61 participants. 85% (34/40) of participants for whom usage data were available, completed all activities, 1 completed five out of six activities, and 5 completed two out of six activities. | 6 and 12 weeks |
| Graham  2020[43] | Primary Care  N=146  USA | IntelliCare platform:  in this study, participants received access to 5 clinically focused apps + Coaching calls, SMS 2-per week, welcome packet via email, 1 onboarding telephone call (30-40 minutes), and the offer for an optional mistreatment call  8 weeks  Each week, a coach recommended a new app to download and try, based on the participant’s preferences and a recommendation protocol. Participants were encouraged to try the newly recommended app but could download any app at any time and use or discontinue apps as preferred  CBT + Positive Psychology  At 8 weeks following treatment, 119/146 (81.5%) had some App use. For all participants, postintervention median time to last app use was 28 days (range 0-212 days) and median days used was 7 (range 0-102 days). | 4, 8, 12, and 16 weeks |
| Guo  2020[44] | Hospital  N= 300  China | Run4Love (Wechat platform):  Two major components: the adapted cognitive-behavioral stress management (CBSM) course (9 sessions), regular physical activity promotions, and automatic progress monitoring (5 phone calls).  12 weeks  NR  CBT (adapted cognitive-behavioral stress management (CBSM) course))  NA | 12, 24 and 36 weeks |

BDI= Beck Depression Inventory; CBT= Cognitive Behavioural Therapy; CES-D= Center for Epidemiologic Studies Depression Scale; NA = not applicable; NR = not reported; PHQ= Patient Health Questionnaire; PRIME-MD= Mental Disorders in Primary Care; STAI= State-Trait Anxiety Inventory. ^$^HARUCard is an intervention used as comparator.

33. Arean, P.A., et al., *The Use and Effectiveness of Mobile Apps for Depression: Results From a Fully Remote Clinical Trial.* J Med Internet Res, 2016. **18**(12): p. e330.

34. Birney, A.J., et al., *MoodHacker Mobile Web App With Email for Adults to Self-Manage Mild-to-Moderate Depression: Randomized Controlled Trial.* JMIR Mhealth Uhealth, 2016. **4**(1): p. e8.

35. Dahne J, Lejuez CW, Diaz VA, Player MS, Kustanowitz J, Felton JW, et al. Pilot Randomized Trial of a Self-Help Behavioral Activation Mobile App for Utilization in Primary Care. Behav Ther. 2019;50(4):817-27

36. Ham, K., et al., *Preliminary Results From a Randomized Controlled Study for an App-Based Cognitive Behavioral Therapy Program for Depression and Anxiety in Cancer Patients.* Front Psychol, 2019. **10**: p. 1592.

37. Ludtke, T., et al., *A randomized controlled trial on a smartphone self-help application (Be Good to Yourself) to reduce depressive symptoms.* Psychiatry Res, 2018. **269**: p. 753-762.

38. Mantani, A., et al., *Smartphone Cognitive Behavioral Therapy as an Adjunct to Pharmacotherapy for Refractory Depression: Randomized Controlled Trial.* J Med Internet Res, 2017. **19**(11): p. e373.

39. Moberg, C., A. Niles, and D. Beermann, *Guided Self-Help Works: Randomized Waitlist Controlled Trial of Pacifica, a Mobile App Integrating Cognitive Behavioral Therapy and Mindfulness for Stress, Anxiety, and Depression.* J Med Internet Res, 2019. **21**(6): p. e12556.

40. Pratap, A., et al., *Using Mobile Apps to Assess and Treat Depression in Hispanic and Latino Populations: Fully Remote Randomized Clinical Trial.* J Med Internet Res, 2018. **20**(8): p. e10130.

41. Roepke, A.M., et al., *Randomized Controlled Trial of SuperBetter, a Smartphone-Based/Internet-Based Self-Help Tool to Reduce Depressive Symptoms.* Games Health J, 2015. **4**(3): p. 235-46.

42. Tighe, J., et al., *Ibobbly mobile health intervention for suicide prevention in Australian Indigenous youth: a pilot randomised controlled trial.* BMJ Open, 2017. **7**(1): p. e013518.

43. Graham, A.K., et al., *Coached Mobile App Platform for the Treatment of Depression and Anxiety Among Primary Care Patients: A Randomized Clinical Trial.* JAMA Psychiatry, 2020. **77**(9): p. 906-914.

44. Guo, Y., et al., *Effect of a WeChat-Based Intervention (Run4Love) on Depressive Symptoms Among People Living With HIV in China: A Randomized Controlled Trial.* J Med Internet Res, 2020. **22**(2): p. e16715.
