# Supplementary material for: Impact of Smartphone App–Based Psychological Interventions for Reducing Depressive Symptoms in People With Depression: Systematic Literature Review and Meta-analysis of Randomized Controlled Trials
Source: JMIR Mhealth Uhealth. 2022 Jan 27;10(1):e29621. doi: 10.2196/29621 (PMC8832272; doi:10.2196/29621)
Supplement: Multimedia Appendix 3 [file mhealth_v10i1e29621_app3.docx]

Multimedia Appendix 3. Quality assessment of included studies

| *Study* | *Risk of bias* | | | | |  |
| --- | --- | --- | --- | --- | --- | --- |
|  | *Randomization* | *Deviation from intended intervention* | *Missing outcome data* | *Measurement of the outcome* | *Selection of the reported result* | *Overall*  *Score* |
| Arean 2016[33] | - | - | - | - | - | Low |
| Birney 2016[34] | - | - | - | - | - | Low |
| Dahne 2018[35] | / | / | - | - | - | Some concerns |
| Ham 2019[36] | - | - | - | - | - | Low |
| Lüdtke 2018[37] | - | - | - | - | - | Low |
| Mantani 2017[38] | - | - | - | - | - | Low |
| Moberg 2019[39] | - | - | + | + | / | High |
| Pratap 2018[40] | - | + | + | - | / | High |
| Roepke 2015[41] | - | - | + | + | / | High |
| Tighe 2017[42] | - | - | - | + | / | High |
| Graham 2020[43] | - | - | / | - | - | Some concerns |
| Guo 2020[44] | - | - | - | + | - | High |

+ High risk of bias; - Low risk of bias; / Some concerns about risk of bias

33. Arean, P.A., et al., *The Use and Effectiveness of Mobile Apps for Depression: Results From a Fully Remote Clinical Trial.* J Med Internet Res, 2016. **18**(12): p. e330.

34. Birney, A.J., et al., *MoodHacker Mobile Web App With Email for Adults to Self-Manage Mild-to-Moderate Depression: Randomized Controlled Trial.* JMIR Mhealth Uhealth, 2016. **4**(1): p. e8.

35. Dahne J, Lejuez CW, Diaz VA, Player MS, Kustanowitz J, Felton JW, et al. Pilot Randomized Trial of a Self-Help Behavioral Activation Mobile App for Utilization in Primary Care. Behav Ther. 2019;50(4):817-27

36. Ham, K., et al., *Preliminary Results From a Randomized Controlled Study for an App-Based Cognitive Behavioral Therapy Program for Depression and Anxiety in Cancer Patients.* Front Psychol, 2019. **10**: p. 1592.

37. Ludtke, T., et al., *A randomized controlled trial on a smartphone self-help application (Be Good to Yourself) to reduce depressive symptoms.* Psychiatry Res, 2018. **269**: p. 753-762.

38. Mantani, A., et al., *Smartphone Cognitive Behavioral Therapy as an Adjunct to Pharmacotherapy for Refractory Depression: Randomized Controlled Trial.* J Med Internet Res, 2017. **19**(11): p. e373.

39. Moberg, C., A. Niles, and D. Beermann, *Guided Self-Help Works: Randomized Waitlist Controlled Trial of Pacifica, a Mobile App Integrating Cognitive Behavioral Therapy and Mindfulness for Stress, Anxiety, and Depression.* J Med Internet Res, 2019. **21**(6): p. e12556.

40. Pratap, A., et al., *Using Mobile Apps to Assess and Treat Depression in Hispanic and Latino Populations: Fully Remote Randomized Clinical Trial.* J Med Internet Res, 2018. **20**(8): p. e10130.

41. Roepke, A.M., et al., *Randomized Controlled Trial of SuperBetter, a Smartphone-Based/Internet-Based Self-Help Tool to Reduce Depressive Symptoms.* Games Health J, 2015. **4**(3): p. 235-46.

42. Tighe, J., et al., *Ibobbly mobile health intervention for suicide prevention in Australian Indigenous youth: a pilot randomised controlled trial.* BMJ Open, 2017. **7**(1): p. e013518.

43. Graham, A.K., et al., *Coached Mobile App Platform for the Treatment of Depression and Anxiety Among Primary Care Patients: A Randomized Clinical Trial.* JAMA Psychiatry, 2020. **77**(9): p. 906-914.

44. Guo, Y., et al., *Effect of a WeChat-Based Intervention (Run4Love) on Depressive Symptoms Among People Living With HIV in China: A Randomized Controlled Trial.* J Med Internet Res, 2020. **22**(2): p. e16715.
